# Development and pilot implementation of guidelines for culturally tailored research recruitment materials for African Americans and Latinos

**DOI:** 10.1186/s12874-022-01724-4

**Published:** 2022-09-24

**Authors:** Jennifer Cunningham-Erves, Sheila V. Kusnoor, Victoria Villalta-Gil, Sarah C. Stallings, Jabari S. Ichimura, Tiffany L. Israel, Paul A. Harris, Consuelo H. Wilkins

**Affiliations:** 1 Department of Internal Medicine, Meharry Medical College, 1005 Dr. D. B. Todd Jr. Blvd., Nashville, TN USA; 2 Center for Knowledge Management, Strategy and Innovation, Vanderbilt University Medical Center, Nashville, TN USA; 3 Department of Biomedical Informatics, Vanderbilt University, Nashville, TN USA; 4 Meharry-Vanderbilt Alliance, Vanderbilt University Medical Center, Nashville, TN USA; 5 Department of Medicine, Vanderbilt University Medical Center, Nashville, TN USA; 6 Vanderbilt Institute for Medicine and Public Health, Nashville, TN USA; 7 Vanderbilt Institute for Clinical and Translational Research, Vanderbilt University Medical Center, Nashville, TN USA; 8 Office of Health Equity, Vanderbilt University Medical Center, Nashville, TN USA

**Keywords:** Cultural tailoring, Minoritized recruitment, Clinical trials, Participant enrollment

## Abstract

**Background:**

Previous studies support cultural tailoring of recruitment materials as a strategy to promote the enrollment of minoritized groups in clinical trials. However, there is a lack of guidance for research teams to create culturally tailored materials, potentially contributing to low recruitment rates of minoritized groups. We describe the development and pilot testing of recruitment material guidelines used to culturally tailor clinical trial recruitment materials targeting African Americans and Latinos.

**Methods:**

The guideline development team consisted of investigators, research staff, and community leaders and members experienced in the recruitment and community engagement of minoritized groups. The recruitment material guidelines were developed using the literature, focus groups with African Americans and Latinos, the teams’ research experience, and guidance from a community advisory board. To assess the effectiveness of the guidelines, a pilot study was conducted comparing advertisement click-through rates and enrollment outcomes between two institutions differing in use of culturally tailored versus non-tailored Facebook banner ads for the “Aspirin Dosing: A Patient-centric Trial Assessing Benefits and Long-Term Effectiveness” (ADAPTABLE) study.

**Results:**

Five themes emerged from focus groups: (1) employ diversity and inclusion in recruitment efforts; (2) access multiple recruitment channels to increase reach and possible participation; (3) increase your “footwork”; (4) personalize outreach and recruitment to specific groups’ beliefs and values; (5) align recruitment messaging with language preferences and motivations for study participation; and (6) specify incentives for participation. Guidelines were: 1) be inclusive; 2) use all forms of media; 3) take a personalized approach; 4) align recruitment messaging with motivations for study participation; 5) specify incentives; and 6) get out into the community. Additional guidelines were developed addressing specific considerations for images and language when targeting African American and Latino populations. Pilot study results demonstrated that clicks per impression ratio (0.47 clicks per impression vs. 0.03 clicks per impression) and the percentage of African American enrollment were significantly higher when using tailored compared to non-tailored ads (12.8% vs. 8.3%, respectively).

**Conclusion:**

The recruitment material guidelines offer practical recommendations to reach diverse populations for clinical trial participation more effectively. Our preliminary data supports use of these guidelines as a strategy to enhance recruitment of minoritized groups into clinical research studies.

**Supplementary Information:**

The online version contains supplementary material available at 10.1186/s12874-022-01724-4.

## Background

In the United States, health disparities across racial and ethnic minoritized groups are well-known and have been extensively documented in the literature [1, 2]. Clinical trials are available to help improve health outcomes through the identification of effective therapies. Yet, these therapies are often insufficiently tested in racial and ethnic minoritized groups due to low clinical trial participation rates [3, 4]. Low participation limits the generalizability of research results, decreases acceptability of treatment options, and impacts the relevance of health outcomes [5–8]. These differences can further exacerbate existing disparities in health outcomes.

Fundamental issues related to recruitment of racial and minoritized groups in clinical trials are well known, but few evidence-based interventions exist to overcome them. The key barriers at the participant level are related to awareness of clinical trials, opportunity to participate, and factors related to acceptance, including distrust of research and the medical community stemming from the long-standing history of mistreatment and abuse such as that encountered by African Americans in the Tuskegee Syphilis Study [9–13]. Similarly, there are obstacles to implementing recruitment strategies including limited resources and expertise to translate or adapt literacy level of documents, limited expertise to culturally tailor recruitment strategies, few, if any, researchers or staff from underrepresented groups, and insufficient long-term relationships with community organizations serving underrepresented racial/ethnic groups [14–16].

Recent reviews of recruitment and retention strategies for minoritized groups support the need for multiple approaches to recruitment because there is “no one-size-fits-all” approach [17–19]. Messaging that has been culturally tailored shows promise in enhancing recruitment of underrepresented groups in research; however, evidence-based guidelines are needed to support the development and use of culturally appropriate recruitment materials and practices. Facebook is now commonly used to recruit participants in research as nearly 69% of US adults report being users [20, 21]. Among the two largest minoritized groups in the U.S., a high percentage of African Americans (74%) and Latinos (72%) reported being users [20]. Furthermore, Facebook allows researchers to advertise to target audiences. This is an ideal platform to use to test the impact of culturally appropriate guidelines with a goal to enhance recruitment of minoritized groups.

With funding from the National Center for Advancing Translational Sciences at the National Institutes of Health, the Recruitment Innovation Center (RIC) was launched in 2016 to help clinical research teams address challenges in recruiting and retaining participants in clinical trials, including enrollment of underrepresented populations [22]. To enhance clinical trial recruitment practices and participation among African Americans and Latinos, we describe: 1) the development of Recruitment Material Guidelines for African Americans and Latinos, and 2) the results from a pilot study assessing the effectiveness of the guidelines.

## Methods

To develop empirically based guidelines to culturally tailor recruitment materials for African Americans and Latinos, we: (1) surveyed the literature to gather insight on recruitment strategies of racial and ethnic minoritized groups in clinical trials; (2) conducted focus groups with African Americans and Latinos to identify ways to improve recruitment materials; (3) developed recruitment material guidelines for minoritized groups with RIC Community Advisory Board (CAB) input; and (4) developed a social media campaign informed by those guidelines and evaluated in a pilot study. Collectively, our study team had expertise in clinical trial recruitment of African Americans and Latinos, which was also used to inform guidelines. This work was approved by the Institutional Review Board (IRB) of Vanderbilt University.

### Phase 1. Review of the literature

We reviewed the literature on the use of cultural tailoring as a strategy to promote recruitment of underrepresented populations in clinical trials. We searched articles indexed in PubMed, CINAHL, and Web of Science up to May 31, 2017, using a combination of controlled vocabulary and text words (see Additional file 1). Articles addressing the effectiveness of using culturally tailored recruitment materials as a strategy for increasing the enrollment of underrepresented populations in clinical trials were included in the review. Results were summarized in a narrative format (see Additional file 1). The results of the literature were used to develop the initial draft of the guidelines.

### Phase II. Focus groups

#### Study design and setting

We used a qualitative, phenomenological study design to better understand the needs and preferences of people who are African American or Latino in research recruitment materials to stimulate interest in research. We further sought to gain input on sample recruitment materials to gain a better understanding of participants’ preferences on recruitment materials. This work was conducted in Middle Tennessee. The COREQ checklist informed the reporting of this study [23]. This component of the study was approved by IRBs of Vanderbilt University and University of Chicago.

#### Sampling and recruitment

A purposive sampling method was used to select participants. Recruitment methods included email and face-to-face invitations sent by community health educators and community program managers of community health centers as well as flyers and newsletters distributed in local community health centers. Additional recruitment mechanisms were ResearchMatch [24] and email listservs at universities and community partners. To further assist in the recruitment of Latinos, community engagement specialists with extensive experience in recruitment of minoritized groups were hired. Inclusion criteria for focus group participants were: 1) English-speaking, Spanish-speaking, or bilingual speaking if Latino; 2) African American or Latino; and 3) age 18 and older. A screening instrument with variables education, age, sex, race/ethnicity, literacy, and internet use was administered to screen participants while ensuring heterogeneity. Participants had the option to self-identify as African American and Latino.

#### Procedures

We conducted six, in-person focus groups in community-based settings (e.g., library, community center, churches) from January to March 2018. Three groups included African American, and three groups included Latinos. Each group lasted 90-min. An extensive, qualitatively trained and experienced PHD-level female researcher (co-author JCE) and a Master’s Level Community Health Educator trained in conducting qualitative focus groups served as the moderators. They were race/ethnicity concordant with participants as past studies suggest that lack of diverse staff could impact research participation and retention among a diverse sample [25]. Members of the research team had existing relationships with the community-based organization leaders in which the community members were recruited. Two members of the research team were present- one took field notes and another handled logistics. Focus groups were standardized with an introduction, followed by a written consent process, a brief survey to obtain additional socio-demographics, and then the discussion. The moderator’s guide was developed by the research team. The discussion was centered on feedback on recruitment materials for a specific study, how the recruitment materials might reach a broader audience, and what would make participants more likely to volunteer for a research study. Questions also asked about impressions of recruitment materials and suggestions for improvement. Participants were compensated a $50 gift card. Focus groups were audio-recorded, transcribed verbatim, and de-identified for analysis. Those conducted in Spanish were transcribed to English. Transcripts were not returned to participants to provide feedback. Survey data were entered into REDCap, a secure, web-based electronic data capture application for building and managing surveys and databases [26].

#### Data analysis and establishing trustworthiness

Descriptive statistics were conducted using SPSS version 25. Two qualitatively trained analysts of NORC at the University of Chicago analyzed the focus group data. Inductive, deductive thematic analysis was used to analyze the data. A hierarchical coding frame was developed based on: 1) a review of the literature on barriers to recruiting minoritized populations for clinical research, and 2) the focus group protocol to identify primary codes related to perceptions of the recruitment materials, preferred recruitment approaches, and the likelihood of clinical trial participation. Using NVivo software, each analyst independently coded the six focus group transcripts. The coding frame was updated based on emerging themes. This was conducted until coding saturation was met. In weekly data review meetings, we reviewed the coded transcripts and discussed emergent themes. Discrepancies were discussed and consensus achieved on key findings. Strategies to ensure trustworthiness were thick, rich descriptions (i.e., a detailed account of experiences and needs of African Americans and Latinos for research recruitment materials), and peer debriefing (i.e., two qualitative data analysts of NORC who were external to the team which promoted data being analyzed unbiasedly).

### Phase III. Development of recruitment material guidelines for African Americans and Latinos with RIC CAB Input

Using the literature and themes from Phase 2, the Recruitment Material guidelines for African Americans and Latinos were developed by a team of investigators, research staff, and community leaders and included members with extensive experience in recruitment and retention and community engagement with minoritized groups. The guidelines were developed using an iterative process and were informed by the literature, the teams’ experience, and focus group feedback. The team met regularly to discuss and rank the guidelines, making recommendations and modifications as needed. Following each meeting, a new iteration was produced while ensuring guidelines were culturally appropriate using linguistic, evidential, socio-cultural, peripheral, and constituent-involving strategies according to Kreuter et al. [27]

A meeting was held with the RIC CAB to present and obtain feedback on the guidelines. The 12-member RIC CAB is a racially, ethnically, and geographically diverse group representing a variety of community-based organizations including health, social service, faith and advocacy and include patients, caregivers, and past clinical trial participants. They have extensive experience in partnering with academic researchers to engage diverse community members as research participants and research partners. Overall, CAB members perceived the recommendations as clear and advised against ranking as they were equally necessary. The CAB suggested grouping the recommendations into themes while merging two of them. Other suggestions were to add or remove specific suggestions for two recommendations to ensure comprehension. A final meeting was held to finalize the recruitment material guidelines for African Americans and Latinos.

### Phase IV. Application of the recruitment material guidelines for African Americans and Latinos

We conducted a comparative study of culturally tailored versus non-tailored recruitment materials and channels using social media on enrollment among African Americans in the ADAPTABLE study. The ADAPTABLE study is a large, pragmatic trial that leverages PCORnet to identify the optimal dose of aspirin for secondary prevention in patients with atherosclerotic cardiovascular disease (ASCVD) [28]. This study was done by embedding our tailored materials into this ongoing study. Specifically, the Duke Clinical Research Institute site of the ADAPTABLE study served as the intervention site where the recruitment materials and channels were adapted using the recruitment material guidelines for African Americans and Latinos, and embedded into their current study in progress. Vanderbilt University Medical Center served as the control site where the recruitment materials were generic. This study was approved by the Institutional Review Boards at Vanderbilt University and Duke University.

#### Development and/or cultural tailoring of recruitment materials and channels of distribution

The marketing and design firm, Redmond based in Memphis, TN, was hired to work with the Recruitment Innovation Center (RIC) to develop marketing materials for each site to be used in a social media campaign. RIC engaged in an iterative process to develop the ads and culturally tailor the existing recruitment materials, including a webpage, flyer, letter, phone script, and recruitment email. Initially, RIC reduced the reading level of the materials from approximately twelfth grade level to seventh grade level.

The second step was the cultural tailoring of the recruitment processes and materials at the Duke site using the recruitment material guidelines for African Americans and Latinos. The newly tailored materials were based on focus group findings and the vast experience of RIC and Redmond team members in recruiting minoritized groups and knowledge was gained through current and previous digital campaign development processes. See Table 1 for sample banner ads for Facebook. The *Redmond* firm iteratively designed the social media campaign. Meetings were held between members of the research team and *Redmond *to discuss changes and proposed additional modifications. Following each meeting, a new iteration of the ads along with campaign plans, media placement, and project reporting were produced. These items were produced using sociocultural, linguistic, peripheral, and constituent-involving strategies [27]. A final meeting was held to ensure the program was culturally appropriate and inclusive of all feedback.

**Table 1:**
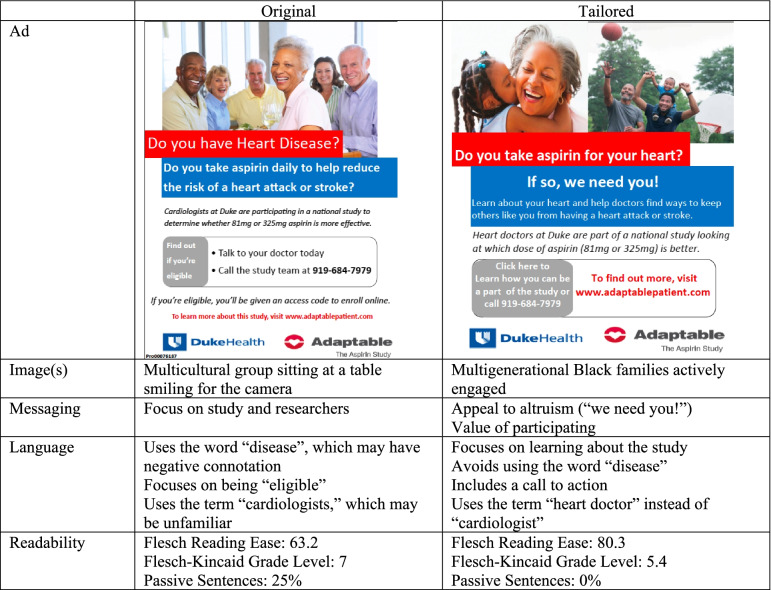
Example of tailored vs non-tailored recruitment banner for Facebook campaign for adaptable study

#### Social media campaign implementation

We implemented a 4-month social media campaign from January 28 through May 15, 2019. African Americans located in the Raleigh-Durham, North Carolina area and Nashville, Tennessee were targeted for recruitment. These areas were selected due to their degree of diversity. Eligibility criteria included being over the age of 40 and African American. The banner ads developed for Facebook could be viewed as impressions on desktop, mobile devices, or tablets. Ads were implemented within a 25-mile radius of each of location. The participants were directed to the study webpage where they could sign up for the study. Table 2 provides the Facebook (FB) population meeting the eligibility criteria and the weekly schedule.

**Table 2:** African American (AA) Population (Pop) on Facebook (FB) and Weekly Impression (Imp) Schedule

Market	FB Pop AA Men 40 +	FB Pop AA Woman 40 +	Weekly Imp Men	Weekly Imp Women
Raleigh/Durham	100,000	160,000	15,000	25,000
Nashville	70,000	110,000	15,000	20,000
Totals	170,000	270,000	30,000	45,000

Note: Impressions refer to the number of times the ads were displayed on a screen

#### Evaluation

There were two forms of data collection to determine the impact of the social media campaign. First *Redmond* monitored the number of clicks or click-through rates on the banner ads. The click-through rates provide the count of actual clicks to the URL page from the banner ad being served. Because the study was embedded into the ADAPTABLE study, the research team of Duke and Vanderbilt monitored and reported the recruitment rates of African Americans at each site. Specifically, recruitment rates were monitored at the Duke site between January 28, 2019 and May 15, 2019 using the culturally tailored banner ad. For the Vanderbilt site, recruitment rates were monitored using the non-tailored ad between February 20, 2019 and April 3, 2019, and then there was a cross-over to use the culturally-tailored ad between April 11, 2019 and May 15, 2019. Descriptives (i.e., frequencies, percentages) were used to determine recruitment rates at each site using SAS software 9.4.

## Results

### Focus groups

There were a total of 64 focus group participants, 29 of whom were African American and 35 of whom were Latino. No participant self-identified as both African American and Latino. All participants completed the focus group. Within the group of participants who were Latino, 71.4% spoke Spanish and 28.6% were bilingual (English and Spanish). Over half the participants in both ethnicity subcategories were from large geographical areas and were female (See Table 3). There was a significant difference in preferred language by race.

**Table 3:** Sociodemographic characteristics and group differences of the participants of the Focus Groups

	Prescreened as African American, allocated to the Focus Groups targeting African American (*N* = 29)	Prescreened as Latino, allocated to the Focus Groups targeting Latino (*N* = 35)	
	%	%	Chi-Sq
Education			
High School or less	24.1	48.6	4.04
College (some college to completion)	55.2	37.1	
Graduate (some graduate to completion)	20.7	14.3	
Preferred Language			
English	100	0	64.00***
Spanish	0	71.4	
Bilingual (English + Spanish)	0	28.6	
Urban/Rural			
Large urban area (> 50.000 people)	82.1	82.8	1.12
Small urban area (2500—50,000 people)	14.3	17.2	
Rural Communities (< 2500 people)	3.6	0	
Gender			
Male	44.8	22.9	3.47
Female	55.2	77.1	
	Mean (SD)	Mean (SD)	t-value
Age	47.75 (16.40)	41.90 (9.73)	1.76

Note: (***) is *p* < .001

Across the focus groups, six broad themes were identified on considerations for recruitment materials and practices to reach diverse populations. We briefly describe each theme below.

#### Theme 1. Employ diversity and inclusion in recruitment efforts

Participants stressed the importance of appealing to diverse audiences through recruitment materials and outreach efforts. Some participants emphasized the consideration of access for non-English speakers and elders, in particular. Among Latino participants especially those who were Spanish speaking, there was emphasis on the need to use the Spanish language. African American participants stated the recruitment materials should reflect and appeal to the languages spoken in an increasing multicultural community. One participant advocated for the inclusion of a telephone number and an internet address on the recruitment materials to meet the needs of the multicultural community members. The participant stated,

*“If I don’t have internet, if I can’t manage the internet, I’m an elder person, but I find a phone number, I can call. “Do you speak Spanish?” “Yes” so there is a Spanish service. You either speak Spanish or English, whatever –. Or they speak both. Yes, but if an African comes, who speaks at least English, not Spanish, but good English. So both should be offered there, with a phone number in which you can be given information, if you don’t or can’t manage the internet.”*

#### Theme 2. Access multiple recruitment channels to increase reach and possible participation

Participants noted that minoritized groups have differential access to information and, by extension, opportunities to participate in a research study or clinical trial. Suggestions were made to use diverse forms of media to reach larger audiences while promoting study participation. Use of electronic communications (email, text messages) and social media (Facebook, YouTube) were commonly mentioned among participants. Traditional media campaigns using newspapers, PSAs on the radio (with “serious” stations), billboards, and television in the areas where the target populations live was also suggested. However, some participants also stressed that some people have limited access to or do not know how to use the internet. Furthermore, some stated that older people may prefer to rely on print and television. One participant offered a multi-pronged approach that integrated multiple outreach strategies. The participant stated,

*“Then, [have] a commercial maybe because you have commercials every day asking for help for research people to come do studies. I see 'em all day on T.V. Come help us out with this study on this medication. If you are having a study in your area, broadcast more. Have some people out on a Saturday at different parts of town where you know there's gonna be people, and you pass this out with someone that speaks very pronounced, like, this is a pamphlet if you would like to have this and help us study research on diseases that we are trying to find a cure for.”*

#### Theme 3. Increase your “footwork”

Participants across groups emphasized a need for a person-centered, yet place-based approach to recruitment. The idea of researchers establishing a relationship and being involved with the community was commonly mentioned. Particularly, researchers need to come to neighborhoods where people reside. One participant said, *“You have to put some foot work into some stuff like this and get it out in your neighborhood, to get the word out, because you got people's family members that's suffering from each one of these diseases that would read upon this and want to participate.”* Participants further suggested stuffing mailboxes and passing out flyers “door-to-door” in places where “everyday” activities occurred in addition to health and medical facilities (e.g., clinics, hospitals, WIC office, pharmacy). These recommendations were based on their personal experiences and knowledge of their communities.

Participants also discussed the need to be creative in distributing materials to reach diverse audiences within a town or city. Placing materials in accessible locations such as flyers on the counter in the “corner” store, in bags at a pharmacy used for prescription pickup, or through a postcard inserted in the daily newspaper were commonly mentioned. Sharing information using posters, flyers, or a bulletin board could occur in libraries, housing facilities including public housing resident meetings, barber shops or beauty shops, places where food is sold (e.g., in stores and restaurants), shopping malls, stores in Latino communities, YMCA, church services, health ministries, community events, health fairs, bus station, and schools and colleges. Overall, participants felt *“the more you put into it, the more you'll get out of it.”*

#### Theme 4: Personalize outreach and recruitment to specific groups’ beliefs and values

Participants also advocated for a proactive, personalized approach to outreach and recruitment. Having someone to talk to in-person about the research was important. Participants stressed the need for telephone hotlines and/or on-site assistance to address questions and enroll subjects. A participant stated, *“I want to talk to somebody who knows what they are talking about and can tell me a reason why I should stay on this phone call.”* Suggestions include having a doctor, nurse, or other recruiter attend a community gathering to talk about the disease and the available clinical trial(s) or holding a workshop to provide counseling or information to address concern on the clinical trial.

The types of messaging to increase personalization was discussed by the participants. Some participants highlighted the use of strength-based messaging. For example, a nurse could state that *"we have a study, we support you, we help you.”* Another option was use of direct appeals. One participant suggested “*set[ting] up a table in a clinic and when you come through the door”*, "*Let me explain this to you and see if you could be a participant.*” One participant stated the recruiter's race or ethnicity should reflect the population targeted. As a recruitment strategy, two Spanish-speaking participants suggested adopting community-oriented, public health prevention approaches that are found in one’s country of origin, where teams of doctors and nurses come to neighborhoods to conduct “*medical workshops”* on an identified health issue.

To reach Spanish speakers, along with other cultural minoritized groups, participants suggested providing options to communicate with someone who is familiar with one’s native language on the phone or in person. One participant observed that at local health fairs there is a lot of information provided, but often the providers or recruiters speak only English. Having multi-lingual recruiters would be beneficial, enabling potential participants to *“feel more confident in approaching them, and for the person to explain it to you, and you’re able to participate.”*

Participants offered insights grounded in their family structure, household composition, and community ties, addressing the role of elders as family leaders and children as culture brokers. By targeting seniors and doing in-person outreach, an African American participant suggested that researchers could reach multiple generations (i.e., children and grandchildren). As this participant noted, *“We still have the hold over them,”* an observation that spoke to the role of elders as a family leader and community gatekeeper. Spanish-speaking participants emphasized sharing information in public schools so that children could bring it home to their parents, as well as providing information in places *“where we go for our kids,”* such as pediatrician offices.

Participants shared that people would accept information from trusted people in their community and places where they felt “*linked*.” African American and Spanish speaking participants noted the primary role of the church in their communities was to be a source of spiritual fellowship, social ties, and information that could provide a *“wealth of people and knowledge.”* In particular, African American participants discussed the importance of connecting with the pastor of the church to reach the membership. Spanish speaking participants suggested providing information during church services in Latino communities. One participant observed, “*If you want people to notice you, go to the church. When the priest finishes mass, before the blessing, the priest allows groups to talk. You can say something quick, and the people who are interested, you tell them, “I’ll see you in the back.” … That’s how it works for people.”* One participant offered that, in general, word of mouth recruitment is strong in Latino communities, emphasizing the importance of trust and personal connections.

#### Theme 5: Align recruitment messaging with language preferences and motivations for study participation

Reflecting on their motivation to participate in a clinical trial, participants across the focus groups stated they had reasons for participation and suggested that messaging on recruitment materials should align with their motivations. Motivations for participation varied across participants. Many stated they *“wanted to help people”,* had a *“willingness to be part of the solution”,* and/or wanted “*to help find a cure*” or “*save lives*”. A participant stated, *“We can be a part of, or can help to find a cure. Many times, we can’t do much for other relatives if they’re going through a problem, but the fact that I can contribute to the cure is very good.”* Participants further reflected on their personal experiences along with those of a family member, friend, or community member who suffered from diseases including cancer, diabetes, kidney disease, sickle cell anemia, or asthma. They stated they would participate to support these individuals. A participant stated, *“You might have a neighborhood with a family member that has the disease, that is suffering one of these diseases, that gets this pamphlet, and be like, "This might help my mom," or "This might help my sister."* Another participant spoke to the value of relating potential participation to one’s family and sense of altruism, saying *"If your loved one has a disease, you have the opportunity to help."* Such an emotional appeal carried greater weight than an impersonal statement from an unknown clinician such as, *“We'd like to have some information from you.”*

Prevention of diseases was another motivating factor. Being aware of family history of disease or susceptibility to disease would motivate participation in a clinical trial. Particularly a few participants suggested use of disease prevalence statistics in the recruitment materials. One participant stated, *“I’m at risk of suffering that disease might make me volunteer.”* Furthermore, a potential benefit of participating in a clinical trial that could be highlighted is the receipt of preventive health care particularly if one does not have health insurance. However, it was noted to address the concern that participation does not cause one to lose health insurance, a fear that negatively impacts recruitment rates. Participants noted the importance of conveying that *“any person can help.”* Knowing *“all types of people”* are valued and can contribute to clinical research would motivate participation, despite differences in language, age, sex, race/ethnicity, religion, and citizenship status.

Motivation to participate was also related to the tone and pitch of the recruitment materials. *“Testimonials of those who have really been able to prevent or have benefitted as a result of their participation in this program”* would motivate participation, as would statistics about the prevalence of a particular disease to increase knowledge. Specific recommendations from the African American participants were to include a call to action, reassure confidentiality, inform participants of potential risk and opportunity for health education, and use a clear and recognizable logo. In terms of language, they stated to avoid negative terminology (i.e., “disease”), use inviting words such as “learn” instead of “join,” and include humor to reach younger people. Latino participants had suggestions on images, including use of medicine-related imagery, research volunteers and scientists, and sensationalized images (e.g., smoking ads). Additional recommendations from the participants were to include testimonials, animation in materials, a Quick Response (QR) code to the study website, and information on the research organization and supporting organizations. In addition, they stated to specify if registration for the study is free. Linguistic suggestions were to include the term “disease” as the first word, include statistics on disease risks in the targeted population, and make the messaging more targeted to “YOU”.

#### Theme 6: Specify incentives for participation

Participants perceived compensation was important and understood that “*each study has different compensation”.* In addition, many participants stated that compensation *“would depend on the type of study”,* conditions, and duration. Being compensated for one’s time and contribution was viewed pragmatically, especially if it involved a long period of time. Participants highlighted that donating one’s time, even if for a few hours, could mean missing out on other income-generating opportunities or personal responsibilities. As one participant noted, *“Well, if y'all are going to put me in the hospital for two weeks, of course I want to be compensated for my time. I mean, I'm missin' part of my life.”* There were two participants in a focus group that were open to participating for free, noting the value of *“learning.”* One participant even stated that learning about the results of the study and whether it was beneficial was an incentive. Among African Americans, a clear description of incentives for participation was emphasized.

Participants reported receiving incentives for taking part in previous [unspecified] studies. Incentives included food, gift cards, and t-shirts. Incentives were identified as motivation for study participation with one participant stating a need for a $100 incentive. However, another participant expressed wariness of an incentive with a high value as that signaled greater risk if involved. The participant stated, *“The bigger the number, the more I feared … we’re talking about $600.00. And I thought, “Wow, what are they going to put in me? Why are they giving me $600.00? What are they going to give me?”.*

### Phase III. Final guidelines

Across the focus groups and the literature, a number of practical suggestions have been identified to improve recruitment methods for minoritized groups and to reach the target populations of interest by suggesting where to place materials and how to reach and motivate people. In addition, health literacy guidelines should also be followed, including use of a 6^th^ to 8^th^ grade reading level, avoiding hyphenated words, and using short words and action verbs [29, 30]. We have summarized the cultural guidelines to develop recruitment materials that target potential participants self-identified as African American or Hispanic/Latino and guidelines specific to each group. See Table 4 for guidelines, application of guidelines, and the cultural area mapped to the guidelines where appropriate using Kreuter et al. [27] cultural-targeting strategies.

**Table 4:** Recruitment material guidelines for African Americans and Latinos using cultural targeting strategies

Guideline	Application of Guideline(s)	Cultural Area Addressed
Use materials inclusive of diverse populations Identify multiple forms of media used the targeted population(s)	-Develop or culturally tailor materials to appeal to targeted populations -Consult members of the target audience to design and/or culturally tailor materials -Provide information in the language of the targeted population -Use diverse forms of media (e.g., print, mass media, digital) based on the local context -Use media sources that reflect socio-cultural standards of targeted population	-Socio-cultural -Socio-cultural/ Peripheral/ Linguistics/Constituent Involving -Social-cultural/Linguistics -Peripheral/ Linguistics -Socio-cultural
Build relationships and show yourself trustworthy	-Offer a phone number for potential participants to call to learn more about the study -Make members of the research team available to the community (e.g., conduct educational sessions, attend community gatherings) -Employ staff who are experienced and/or trained in engaging diverse ethnic/racial groups -Use family-centered recruitment materials and practices -Provide information in the language of the targeted population -Invest time to build personal connections with communities -Personalize recruitment materials by addressing them to the potential participant	-N/A -Socio-cultural -Socio-cultural -Socio-cultural -Socio-cultural/Linguistics -Socio-cultural -Socio-cultural
Align messaging with motivations for study participation	-Appeal to altruistic principles (e.g., describe benefits to society, family members, and the community) -Provide general information about health risks rather than information about disparities specific to the target population -State that everyone is valuable for research	-Socio-cultural -Evidential -Linguistics
Specify incentives for study participation	-Explain incentives and responsibilities -Provide information about non-monetary incentives (e.g., return of results, learning about the disease) -Pragmatically calculate monetary incentives (e.g., transportation costs, time off work)	-Linguistics -Evidential -N/A
Take material to community locations used by the targeted populations	-Go door-to-door and place recruitment materials -Place materials in locations that are easy access and community transited (e.g., supermarket, church) -Do community outreach by providing materials in places targeted audiences convene	-N/A -N/A -N/A
**African Americans Only**		
Use preferred terminology of the targeted population	-Avoid using terms with a negative connotation such as “disease” -Use inviting words such as “learn” instead of “join” -Include humor to reach younger people	-Linguistics -Linguistics -Linguistics
Use visuals recognized by the targeted population	-Show a clear and recognizable logo of academic institutions	-Peripheral
Address informational needs specific to the targeted population Specify incentives for study participation	-Include a call to action -Reassure confidentiality -Clearly state potential risks and benefits -Clearly describe incentives to weigh risks and benefits of study participation	-Linguistics -Linguistics -Evidential / Linguistics -Linguistics
**Latinos Only**		
Use visuals recognized by the targeted population	-Use images related to medicine or scientists -Use images of research participants and their testimonials -Use sensationalized images (e.g., smoking ads) -Include animation (where applicable)	-Peripheral -Peripheral -Peripheral -Peripheral
Use preferred terminology of the targeted population	-Make the messaging more targeted to “YOU” (i.e., use second person when referring to the potential participant) -Put the disease as the first word on material to motivate participation	-Linguistics -Linguistics
Address informational needs specific to the targeted population	-Provide QR code for study website -Include information about risks of participation to instill confidence to participate -Include statistics about the diseases -Indicate costs to participate in clinical trial -Include information about the research organization and its supporting organizations, if applicable	-Peripheral -Linguistics -Linguistics -Linguistics -Linguistics

### Phase IV. Pilot study

**Fig. 1:**
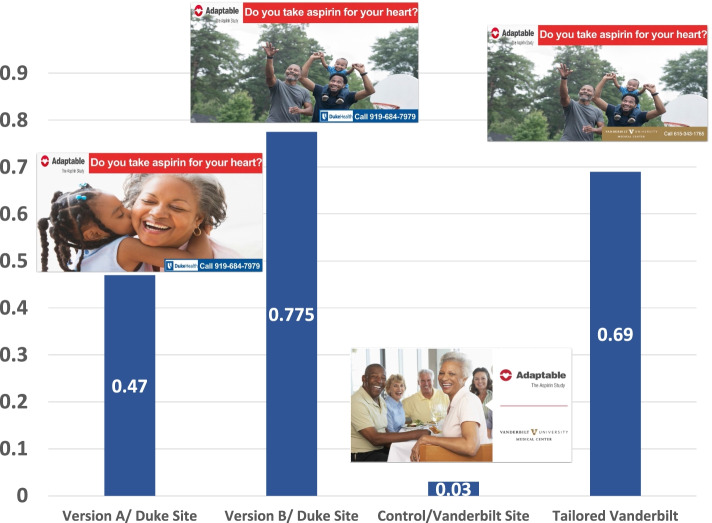
Clicks per Impression on Facebook Ads for Duke Site versus Vanderbilt Site

Clicks per Impression Ratio on the tailored (i.e., Duke site) were significantly higher compared to non-tailored ads (i.e., Vanderbilt University Medical Center). See Fig. 1. At the Duke site, 117 participants were enrolled from January 28, 2019 to May 15, 2019. The majority were white (n = 98), followed by African American (n = 16), other (n = 1) and unknown (n = 2). At the Vanderbilt University Medical Center site, 60 enrolled (55 White, 5 African American) from February 20 to April 3, 2019 using the non-tailored ad compared to 58 enrolled from April 11 to May 15, 2019 (52 White, 6 African American) using the tailored ad. Specifically, 12.8% of African Americans enrolled at the Duke site (i.e., the intervention site) compared to 8.3% at Vanderbilt University Medical Center (i.e., control site).

## Discussion

Increasing clinical trial participation of minoritized groups is necessary [31], and the development of cultural-tailoring recruitment guidelines demonstrates promise [32]. We developed culturally appropriate, recruitment material guidelines for minorized groups using a multi-layered approach. The literature related to the application of cultural targeting strategies to develop recruitment materials served as the foundation for guideline development integrated with input from people who are African American and Latino on strategies to increase reach of recruitment materials in clinical trials. Collectively, this allowed us to develop guidelines which were deeply rooted within the African American and Latino cultures.

Studies continue to explore strategies to increase recruitment and retention across minoritized groups, including the impact of cultural tailoring [17–19]. For example, a study by Huffman and colleagues [32] found that use of sociocultural recruitment mediums, including culturally relevant advertisements, community partnerships, and sociocultural events (such as events involving Black History Month) were more effective than non-sociocultural mediums in promoting interest, as assessed by scheduling a baseline visit, in a weight-loss intervention study. Similar to past studies [33], we found that African Americans and Latinos had a preference for diverse, yet tailored recruitment strategies and multiple channels for disseminating recruitment materials. Furthermore, they emphasized the need to specify incentives, an emerging theme in the literature [34]. Of interest, participants highlighted the need and strategies for researchers and their teams to directly interact with communities. Similar findings have been seen across the literature for different levels of the research process [35]. The emerging findings for the recruitment materials to align with motivations for research participation suggest the need to have a clear understanding of the target audience, their culture, and reasons for participation. Our findings did yield distinctions in needs for recruitment between African Americans and Latinos. For example, African Americans requested a logo on recruitment, which could reflect their need to identify if the source is trustworthy. This is an important concept as mistrust and distrust in research is one of the most cited barriers to research participation [9–13]. Among Latinos, a need for a variety of imagery on materials could reflect the language barriers and might increase comprehension to influence decision-making around research participation [36].

Past research has yielded mixed effects on the application of culturally targeted recruitment strategies on clinical trial recruitment rates. For example, while one study found that use of ethnically targeted statements in a direct mail recruitment letter resulted in increased response rate of minoritized women [37], another study reported that tailoring recruitment letters with racial/ethnic-specific information about health risks and disparities did not impact screening or enrollment [38]. A variety of factors may underlie differences in outcomes observed in studies evaluating the impact of culturally tailoring recruitment materials, including population differences and inconsistencies in how cultural tailoring was applied to deeper dimensions of culture [27]. To the best of our knowledge, we are the first to develop cultural tailoring guidelines for recruitment materials and to assess the impact of applying the guidelines on recruitment in a clinical trial. We chose to use the Facebook platform to test the recruitment guidelines for African Americans and Latinos based on previous studies supporting use of the platform as a recruitment tool to reach diverse audiences within specified geographic areas [39–41]. In our sample, we observed an increase in recruitment rates among African Americans who received the culturally targeted recruitment materials. These findings suggest culturally tailored recruitment materials for African Americans and Latinos based on these guidelines could increase recruitment rates among African Americans. Our study further supports Facebook as a channel of communication to promote the health of communities, and in our case, to increase participation in clinical trials among African Americans. Future studies should explore application of these guidelines in a larger sample and across minoritized groups and research programs.

### Strengths and limitations

A major strength of this study is the application of cultural targeting approaches to develop the recruitment material guidelines for African Americans and Latinos. Engaging the community to advocate for their needs around research recruitment informs researchers on how to approach the community for research participation, potentially a trust-building strategy between researchers and underrepresented research groups like African Americans and Latinos [42]. Another strength is this was a multi-site study which increases generalizability [43]. Limitations do exist in this study. While our guidelines were developed for minoritized groups, specifically African Americans and Latinos, our pilot study only tested these guidelines among African Americans. This reflects the ADAPTABLE study team not having the capacity to respond to individuals who did not speak English. A future study is needed to explore application of these guidelines within studies seeking to recruit Latino community members. Second, our pilot study tested these guidelines on an online platform, which can cause a “digital divide” (especially in terms of internet access) [44], that would affect the targeted groups that could benefit the most. Third, it is hard to determine if the change in recruitment rates were solely due to the implementation of culturally tailored ads; however, use of the control group increases the likelihood. Last, because our study was embedded into an ongoing study, we were limited in terms of the recruitment strategies used as well as the data obtained on each participant.

## Conclusion

We developed guidelines for recruitment materials for African Americans and Latinos using cultural-targeting strategies. We identified areas to improve acceptability among African Americans and Latinos, and effectiveness of recruitment materials among African Americans. These guidelines can be applied by others to inform the development of recruitment materials aiming to enhance recruitment of other minoritized groups. This could ultimately increase diversity in research participation and potentially improve health outcomes.

## Supplementary Information

**Additional file 1.** Narrative review of the effectiveness of culturally tailoring recruitment materials as a strategy for increasing the enrollment of underrepresented populations in clinical trials.

## Data Availability

The data generated and/or analyzed during the current study have not been made publicly available in order to protect participants’ privacy and confidentiality. The focus group transcripts, while deidentified, may still contain information that could be used to link participants’ comments to their identities and under Vanderbilt University Medical Center’s External Data Sharing Policy are considered Human data with Individually Identifiable Health Information or Research Health Information. As such, they cannot be distributed to external entities without a data sharing agreement and participant consent. Summarized data are however available from the corresponding author upon reasonable request.

## Abbreviations

ADAPTABLE: Aspirin Dosing: A Patient-centric Trial Assessing Benefits and Long-Term Effectiveness
ASCVD: Atherosclerotic cardiovascular disease
CAB: Community Advisory Board
FB: Facebook
IRB: Institutional Review Board
NCATS: National Center for Advancing Translational Sciences
NIH: National Institute of Health
RIC: Recruitment Innovation Center
