# Supplementary material for: Development and pilot implementation of guidelines for culturally tailored research recruitment materials for African Americans and Latinos
Source: BMC Med Res Methodol. 2022 Sep 24;22:248. doi: 10.1186/s12874-022-01724-4 (PMC9508728; doi:10.1186/s12874-022-01724-4)
Supplement: Supplementary file 1 — Additional file 1. Narrative review of the effectiveness of culturally tailoring recruitment materials as a strategy for increasing the enrollment of underrepresented populations in clinical trials. [file 12874_2022_1724_MOESM1_ESM.docx]

**Additional file 1.** Narrative review of the effectiveness of culturally tailoring recruitment materials as a strategy for increasing the enrollment of underrepresented populations in clinical trials

**Methods**

The PubMed, CINAHL, and Web of Science databases were searched up to May 31, 2017, using a combination of controlled vocabulary and text words, for articles addressing use of cultural tailoring as a strategy to promote recruitment of underrepresented populations in clinical trials. The searches focused on three concepts. Concept 1 included the following terms: Latino, Latina, African American, African Americans, Hispanic American, Hispanic Americans, Mexican American, Mexican Americans, blacks, Spanish speaking, minority groups, hard to reach, black population, Hispanic population, Spanish speaking, minority population, vulnerable, high risk, high crime, low income, and poverty. Concept 2 included the terms cultural competency, cultural competence, culturally tailored, culturally sensitive, culturally appropriate, culturally mindful, cultural tradition, cultural diversity, culture sensitive, cultural sensitivities, cultural sensitivity, and culturally appropriate. Concept 3 included the terms patient selection, research subjects, recruitment, recruiting, retention, retaining, enrolling, enrollment, enrolment, barrier, barriers, challenge, challenges, overcome, overcoming, difficulty, and difficulties. Articles addressing the effectiveness of using culturally tailored recruitment materials as a strategy for increasing the enrollment of minoritized groups in research were included in the review, and the results were summarized using a narrative format.

**Results**

The search of the literature identified six studies evaluating the impact of tailoring recruitment materials as a strategy for increasing the enrollment of underrepresented populations in clinical trials (Bowers et al., 2017; Brown et al., 2012; Brown et al., 2015; Huffman et al., 2016; Kiernan et al., 2000; Satia et al., 2005). Of the six studies identified through the review, three reported a significant impact of cultural tailoring (Bowers et al., 2017; Brown et al., 2012; Huffman et al., 2016) on either hypothetical willingness to participate (Bowers et al., 2017) or participation in a clinical trial (Brown et al., 2012; Huffman et al., 2016). The successful strategies included use of a low-income, racially, and ethnically diverse community advisory board to review and revise recruitment materials (Bowers et al., 2017), use of ethnically-targeted statements in a direct mail letter (Brown et al., 2012), and use of sociocultural mediums (Huffman et al., 2016). See below for a summary of studies reporting the impact of cultural tailoring that were identified in the review:

**Studies reporting successful use of cultural tailoring to enhance recruitment:**

Bowers and colleagues (2017) evaluated the impact of using feedback from a low-income, racially and ethnically diverse community advisory group to develop recruitment materials. The study compared reactions and hypothetical willingness to participate among two groups of participants: Group A viewed standard recruitment materials (n=55); and Group B viewed recruitment materials after review by a community advisory group (n=45). The revisions included shortening the document, adding colorful graphics, simplifying the language, using bulleted lists and charts, and adding more white space. The study found that participants in Group B were more likely to report that the material was easy to understand and that they would participate in the study than those who viewed the standard materials (Group A).

Brown and colleagues (2012) evaluated the impact of use of personalization and ethnically-targeted statements in a direct mail recruitment letter as a strategy for promoting the recruitment of minority women in a weight management trial. The study used a 2 X 2 factorial design to test the impact of personalization (non-personalized vs. personalized) and statement (generic vs. ethnically-targeted). The study found that the use of an ethnically-targeted statement increased the response rate; however, personalization did not have an impact.

Huffman and colleagues (2016) examined the impact of using recruitment strategies that specifically targeted African Americans (sociocultural mediums) vs. general strategies (non-sociocultural mediums) on enrollment in a weight-loss trial. The study found that use of sociocultural mediums increased the likelihood that families were scheduled for a baseline visit, and that of those who were enrolled, families who were recruited with the sociocultural mediums were more likely to have lower income and less likely to be employed.

**Studies reporting a lack of impact of cultural tailoring on recruitment of minoritized groups:**

Brown and colleagues (2015) examined the impact of tailoring recruitment letters with information about health risks and disparities according to racial/ethnic group on screening and enrollment in a preventive lifestyle intervention trial targeting women at risk for diabetes. Participants were randomized to receive either the tailored or standardized letter. The letters were written in both English and Spanish. The study found that tailoring the recruitment letters did not impact screening or enrollment rates; however, there was a trend towards increased screening and enrollment among Latina women who preferred Spanish, and a trend towards lower enrollment among Latina women preferring English.

Kiernan and colleagues (2000) evaluated the impact of tailoring a direct mail recruitment flyer regarding a dietary intervention study on response rate. Three versions of the flyer were prepared: 1) non-personalized flyer without risk information (control); 2) personalized, hand-signed flyer with cardiovascular risk information for the general public; 3) personalized, hand-signed flyer with risk information for Hispanics. The study found that the response rate was significantly higher with use of the personalized, hand-signed flyer with cardiovascular risk information for the general public compared to the control flyer. However, the response rate was not significantly higher when risk information specific to Hispanics was included compared to the version with general risk information.

Satia and colleagues (2005) evaluated strategies for recruiting African Americans in a cancer surveillance study. Potential enrollees were randomly allocated to receive either: 1) a generic letter describing the study purpose and assurance of confidentiality, 2) a culturally sensitive letter, which included the principal investigator’s photo to show that she is African American, information about the need for data on African Americans, and the potential benefits toothers, 3) a culturally sensitive letter with study compensation information, 4) a generic letter with an incentive, or 5) a culturally sensitive letter with an incentive. The study found that incentive-based strategies resulted in increased participation, and cultural-based strategies had little impact.

**Conclusions:**

Evidence regarding the effectiveness of cultural tailoring to enhance minority recruitment is limited. While some studies observed a significant impact of cultural tailoring, others observed no effect. None of the studies identified in the review reported guidelines for developing culturally tailored materials.

**References:**

Bowers B, Jacobson N, Krupp A. Can Lay Community Advisors Improve the Clarity of Research Participant Recruitment Materials and Increase the Likelihood of Participation? Res Nurs Health. 2017 Feb;40(1):63-69. doi: 10.1002/nur.21752. Epub 2016 Sep 30. PMID: 27686332; PMCID: PMC5632932.

Brown SD, Lee K, Schoffman DE, King AC, Crawley LM, Kiernan M. Minority recruitment into clinical trials: experimental findings and practical implications. Contemp Clin Trials. 2012 Jul;33(4):620-3. doi: 10.1016/j.cct.2012.03.003. Epub 2012 Mar 16. PMID: 22449836; PMCID: PMC3361553.

Brown SD, Partee PN, Feng J, Quesenberry CP, Hedderson MM, Ehrlich SF, Kiernan M, Ferrara A. Outreach to diversify clinical trial participation: A randomized recruitment study. Clin Trials. 2015 Jun;12(3):205-11.

Huffman LE, Wilson DK, Kitzman-Ulrich H, Lyerly JE, Gause HM, Resnicow K. Associations between Culturally Relevant Recruitment Strategies and Participant Interest, Enrollment and Generalizability in a Weight-loss Intervention for African American Families. Ethn Dis. 2016 Jul 21;26(3):295-304.

Kiernan M, Phillips K, Fair JM, King AC. Using direct mail to recruit Hispanic adults into a dietary intervention: an experimental study. Ann Behav Med. 2000 Winter;22(1):89-93.

Satia JA, Galanko JA, Rimer BK. Methods and strategies to recruit African Americans into cancer prevention surveillance studies. Cancer Epidemiol Biomarkers Prev. 2005 Mar;14(3):718-21.
